# Supplementary figures and images for: Real-time imaging of mitochondrial redox reveals increased mitochondrial oxidative stress associated with amyloid β aggregates in vivo in a mouse model of Alzheimer’s disease
Source: Mol Neurodegener. 2024 Jan 18;19:6. doi: 10.1186/s13024-024-00702-2 (PMC10797952; doi:10.1186/s13024-024-00702-2)

Supplementary Figure 1.

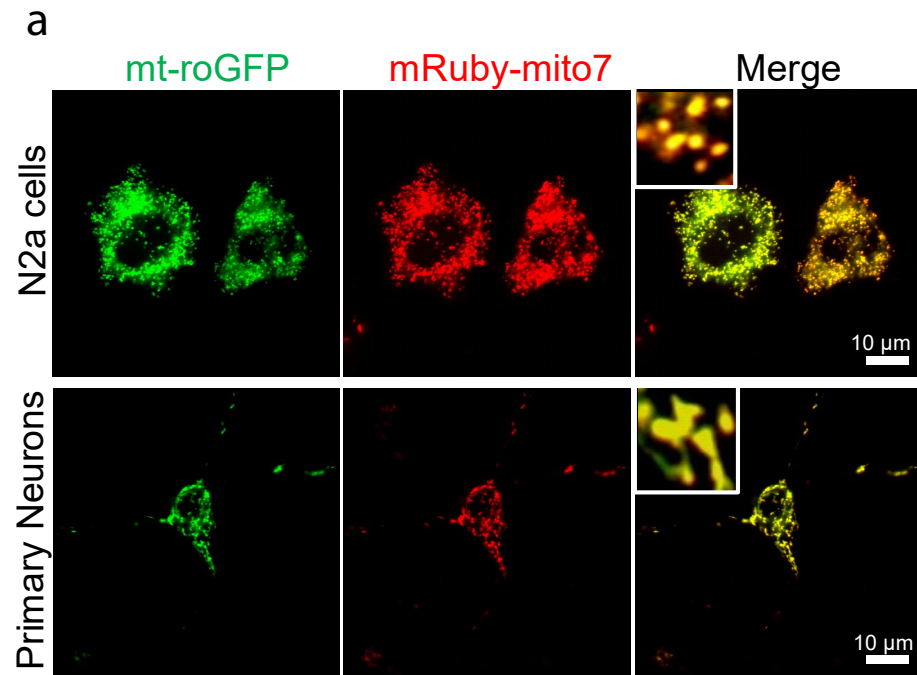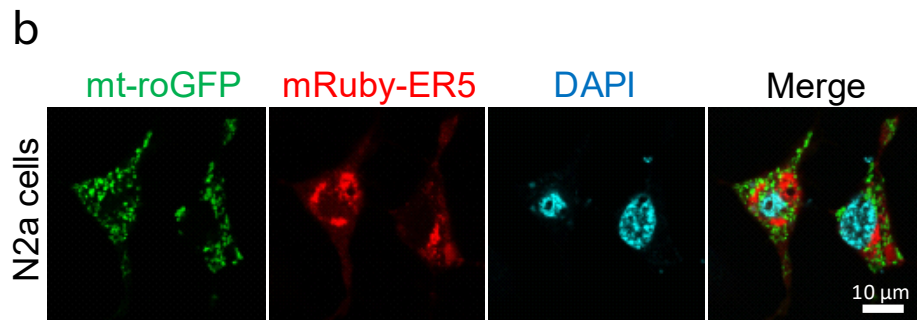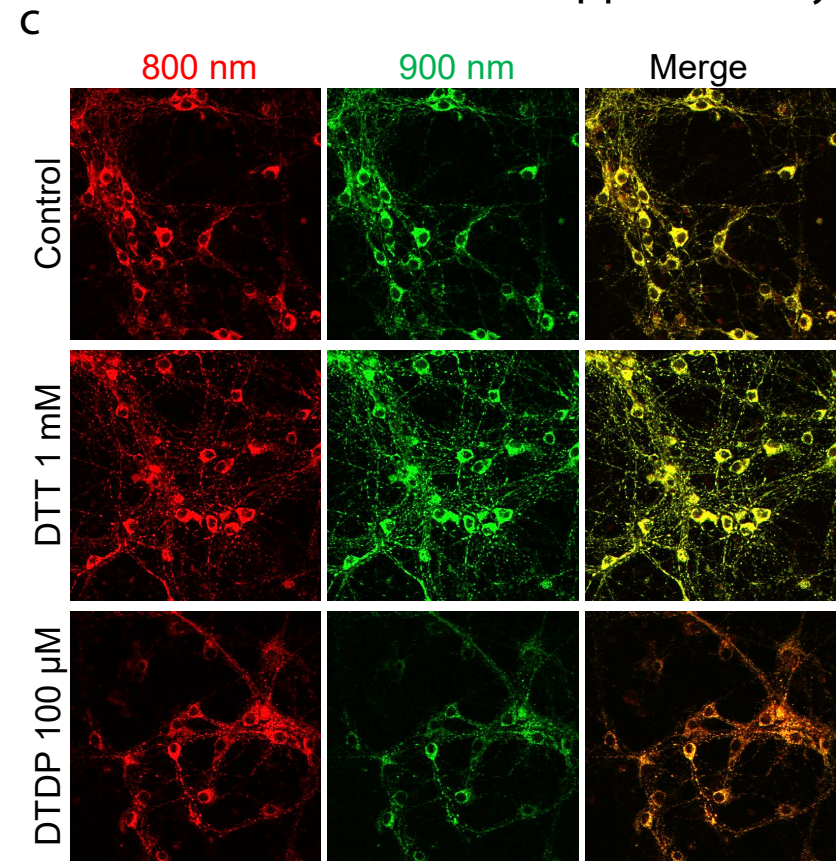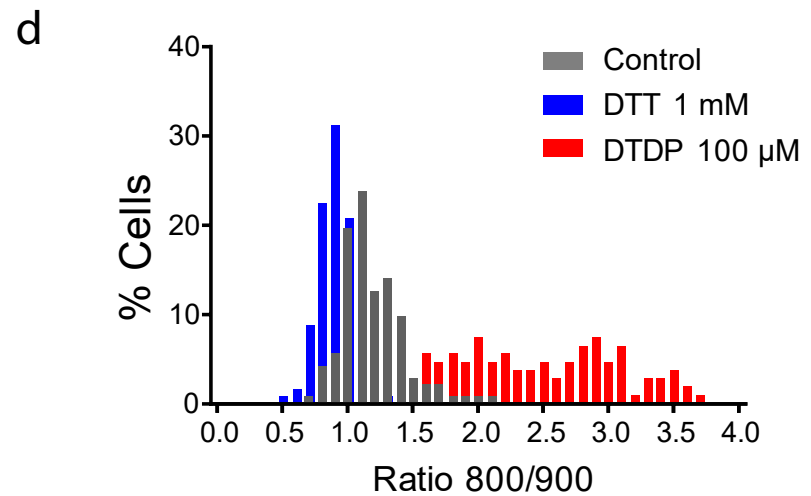

a

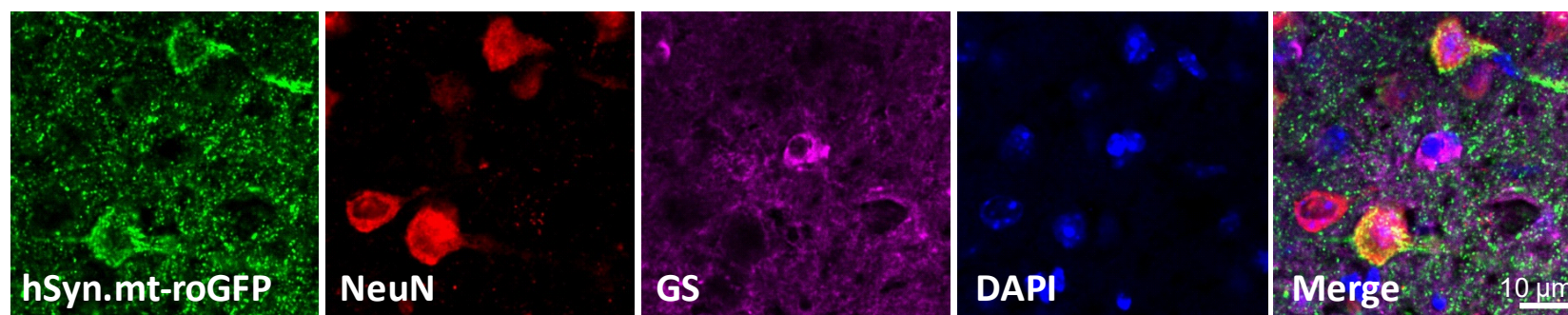

b

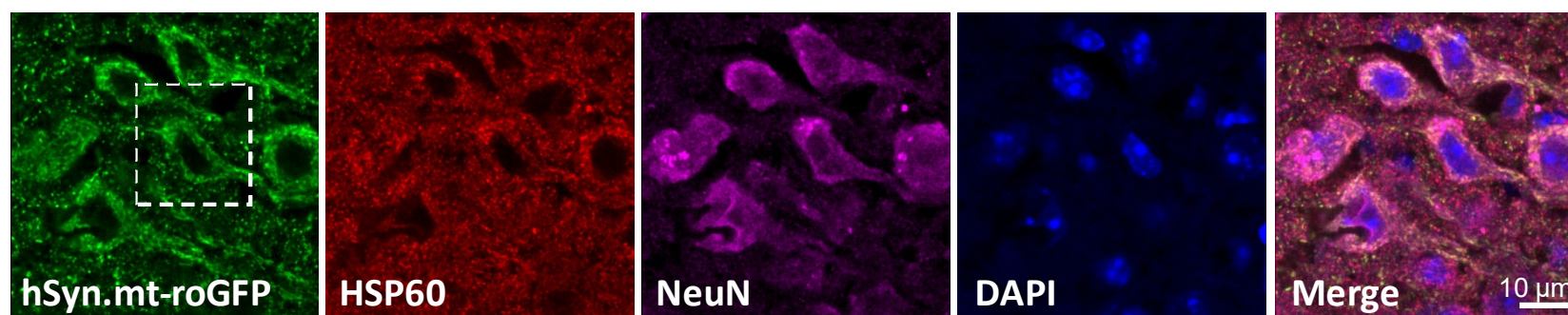

c

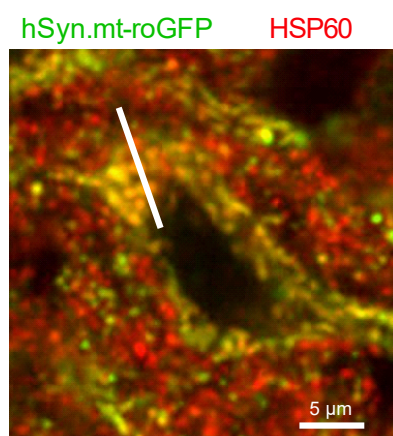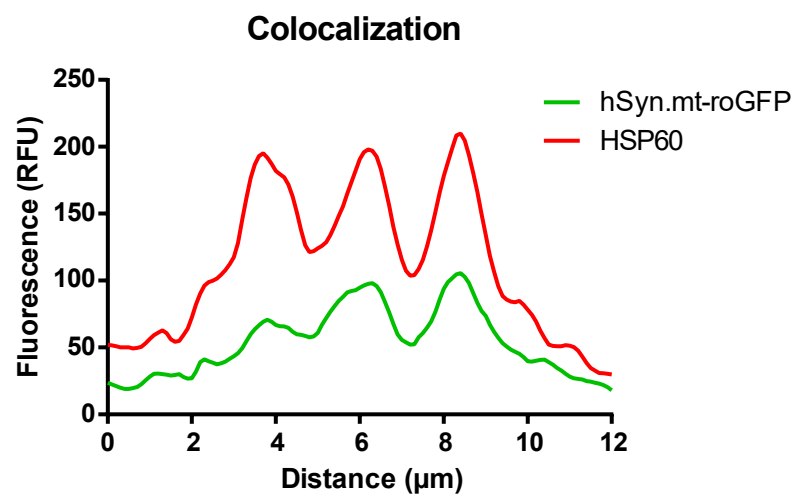

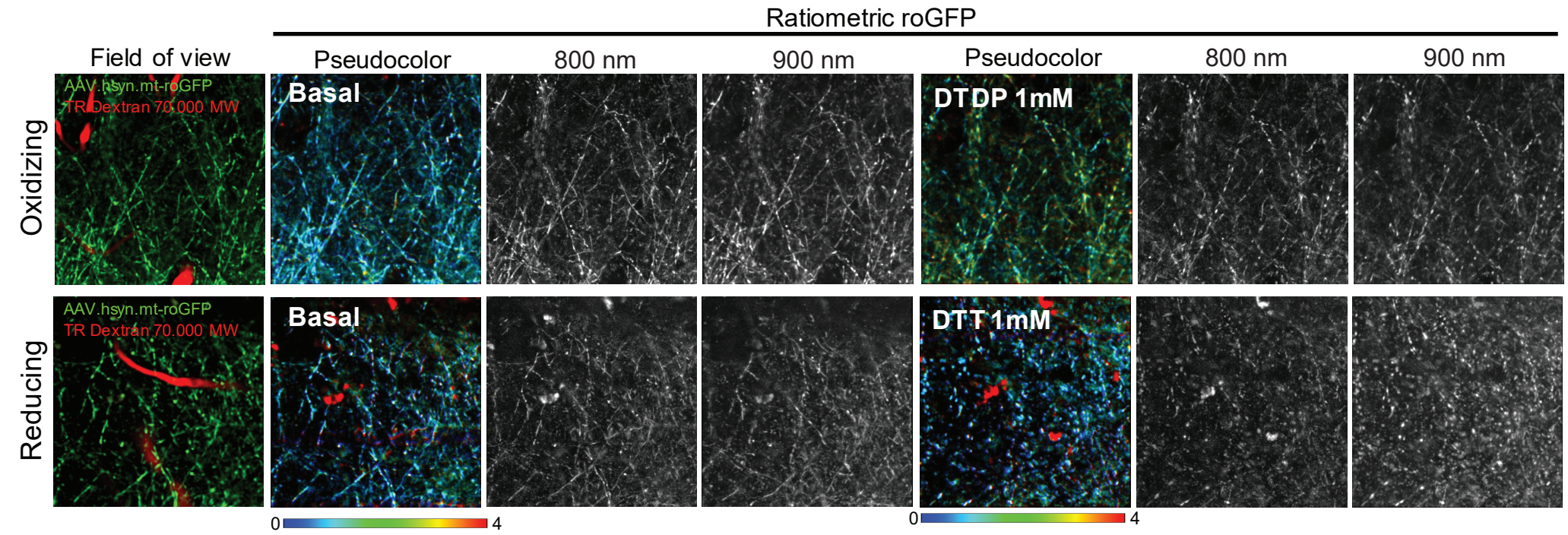

# Non-Tg

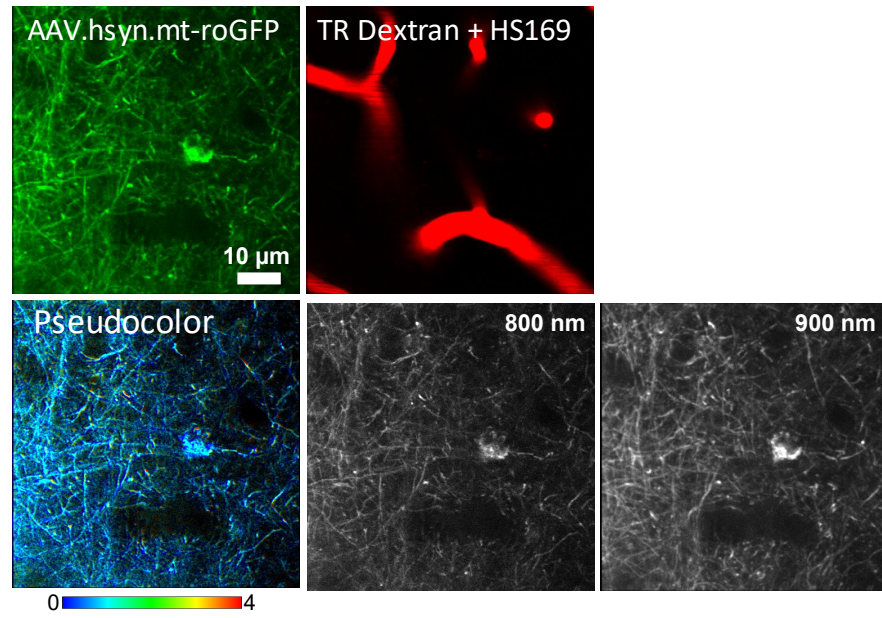

# APP/PS1

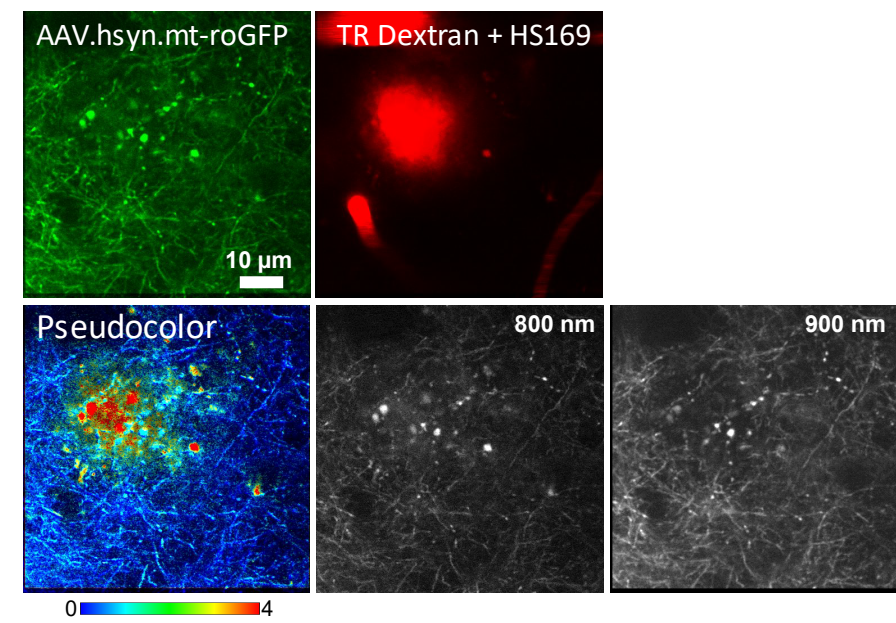

a

Males

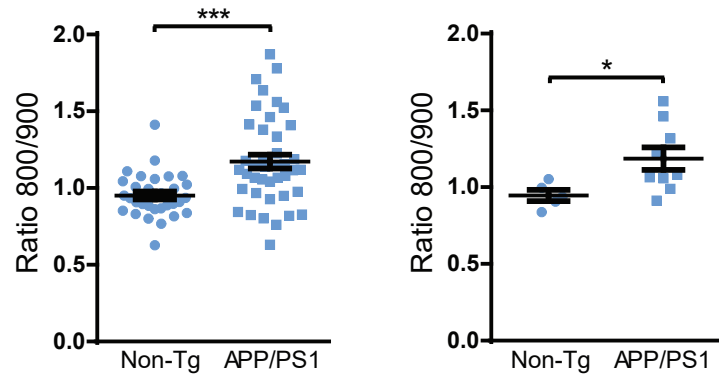

b

Females

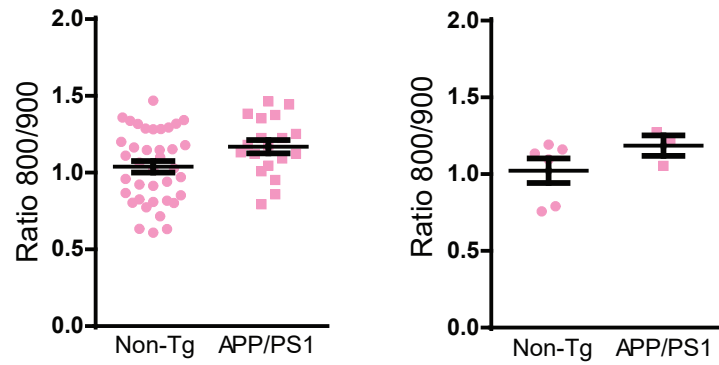

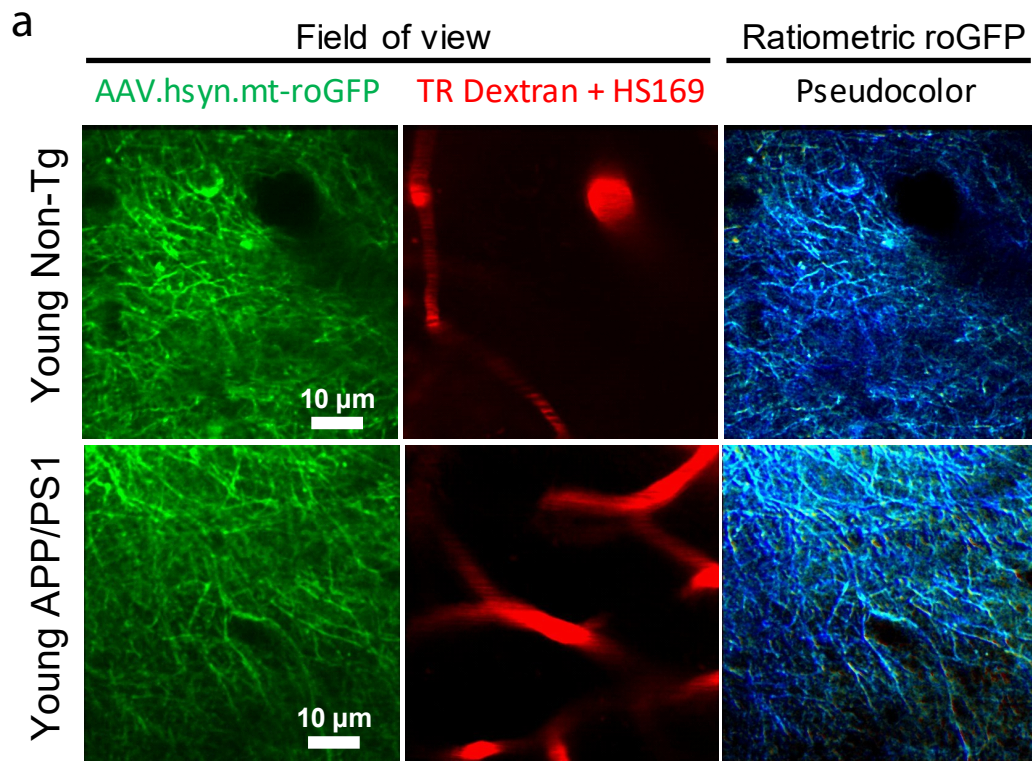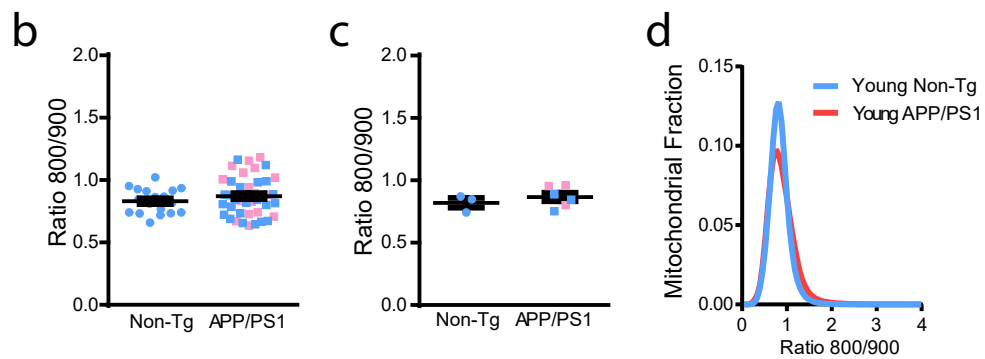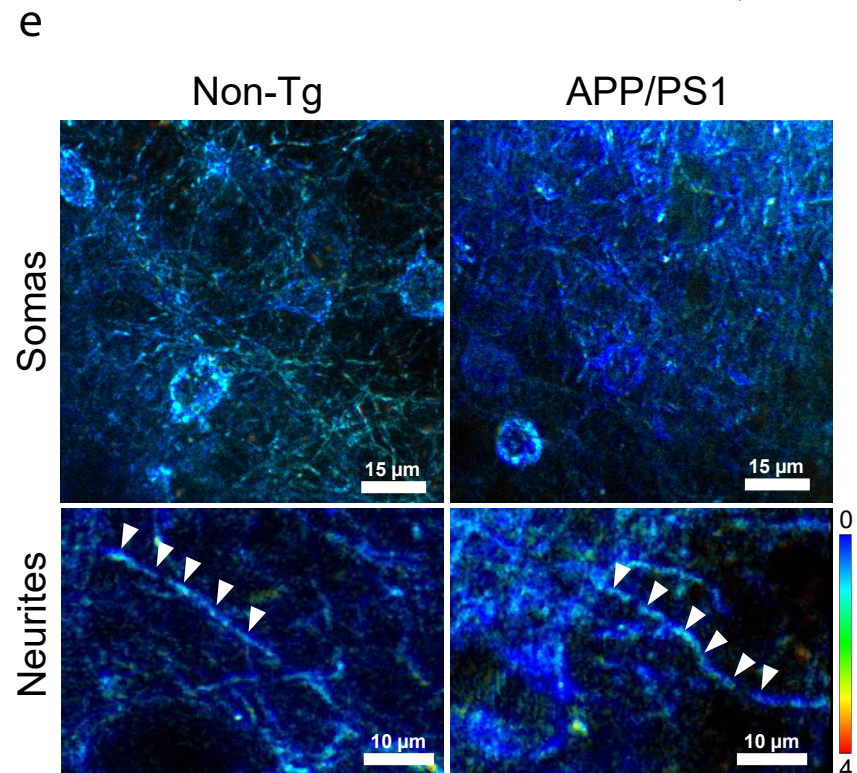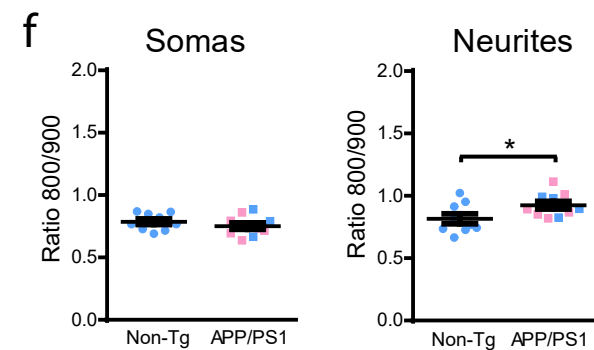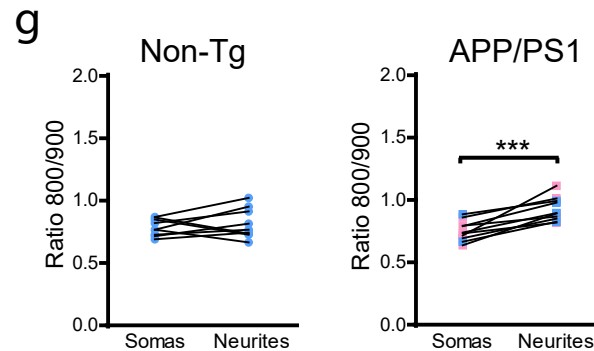

Young Non-Tg

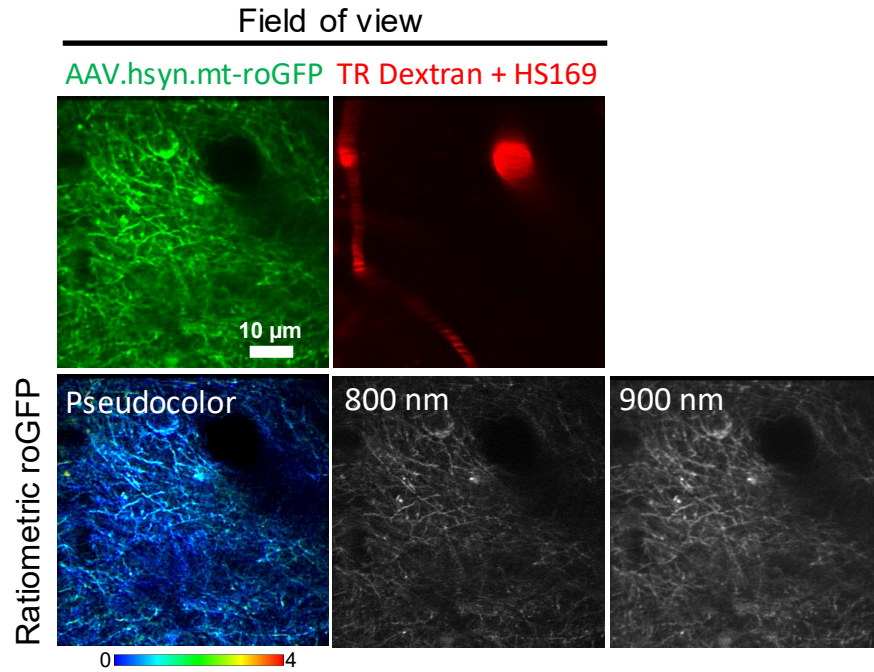

Young APP/PS1

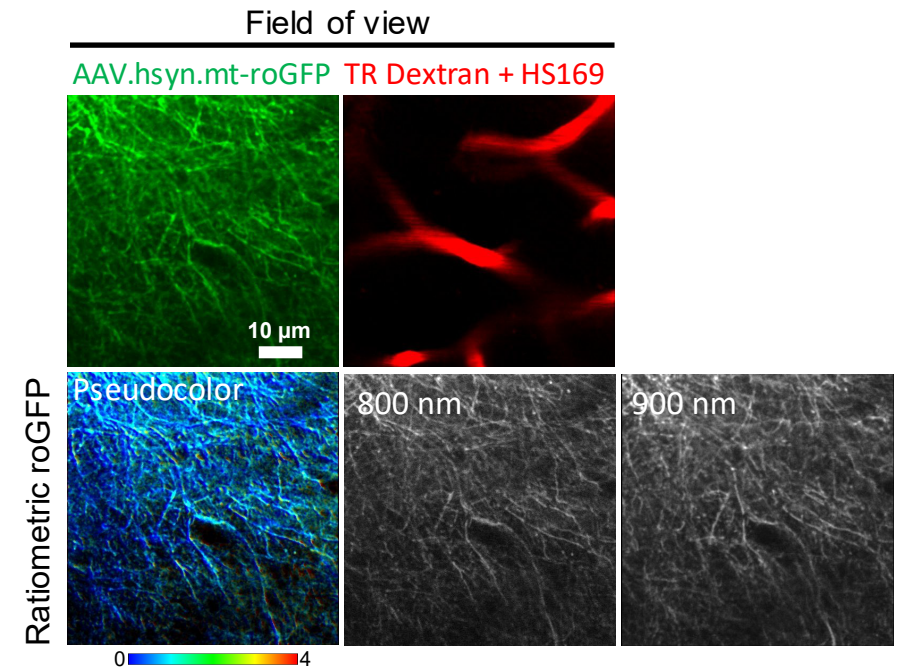

3b

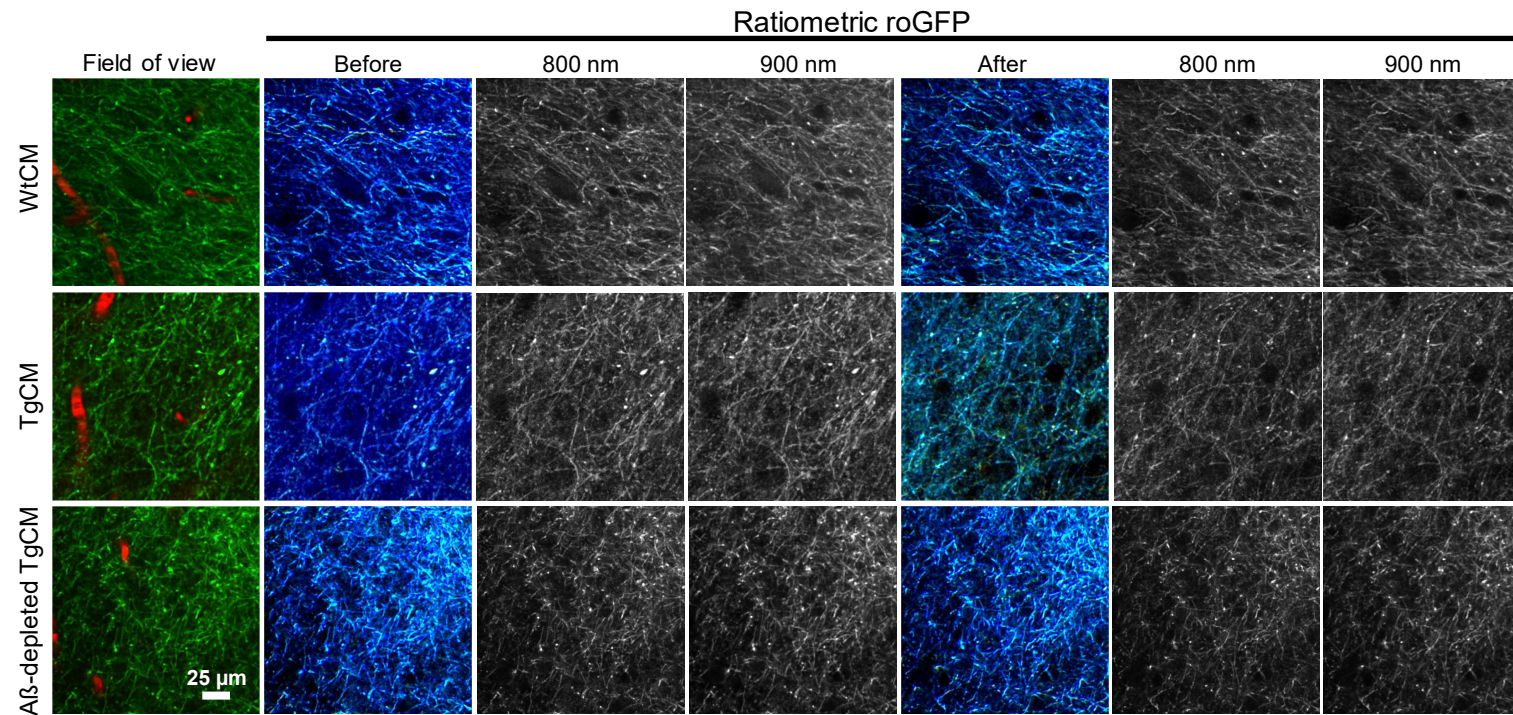

4a

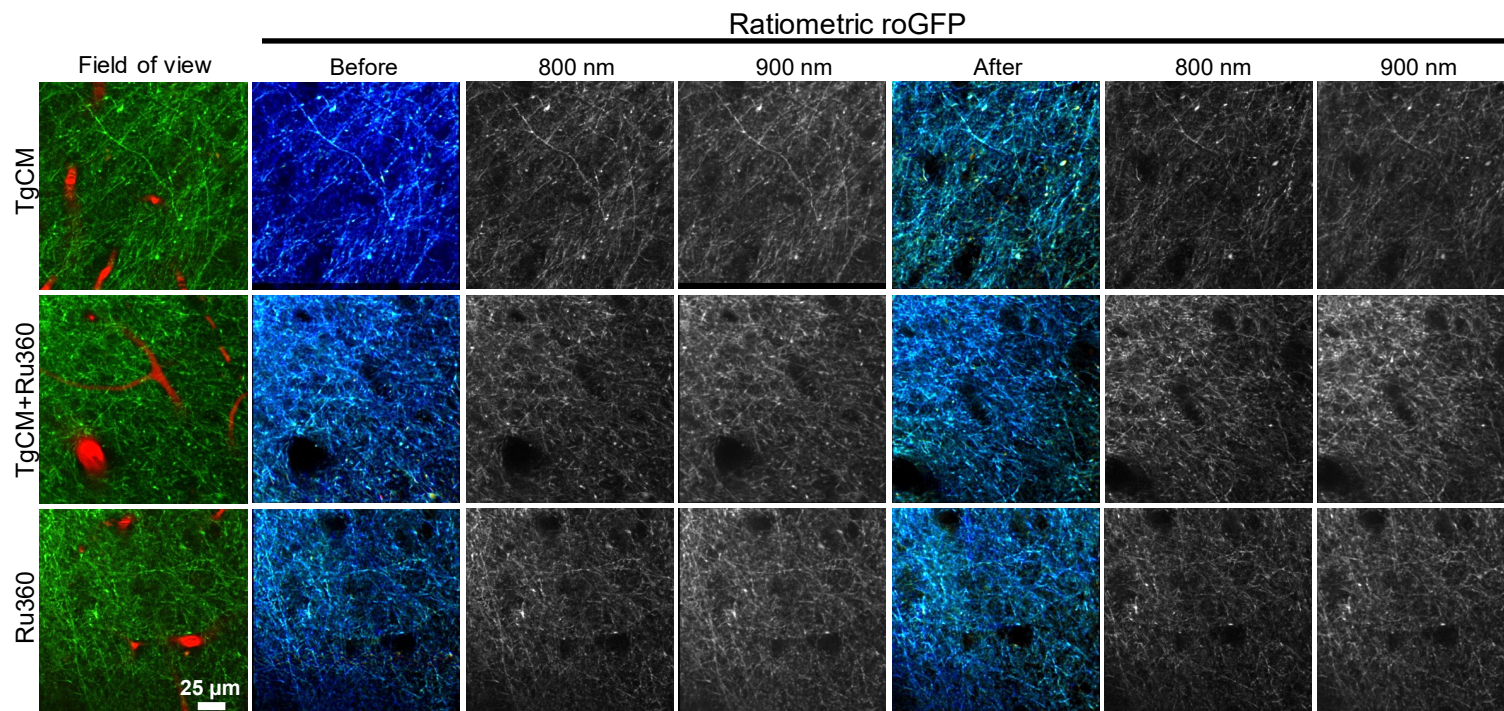

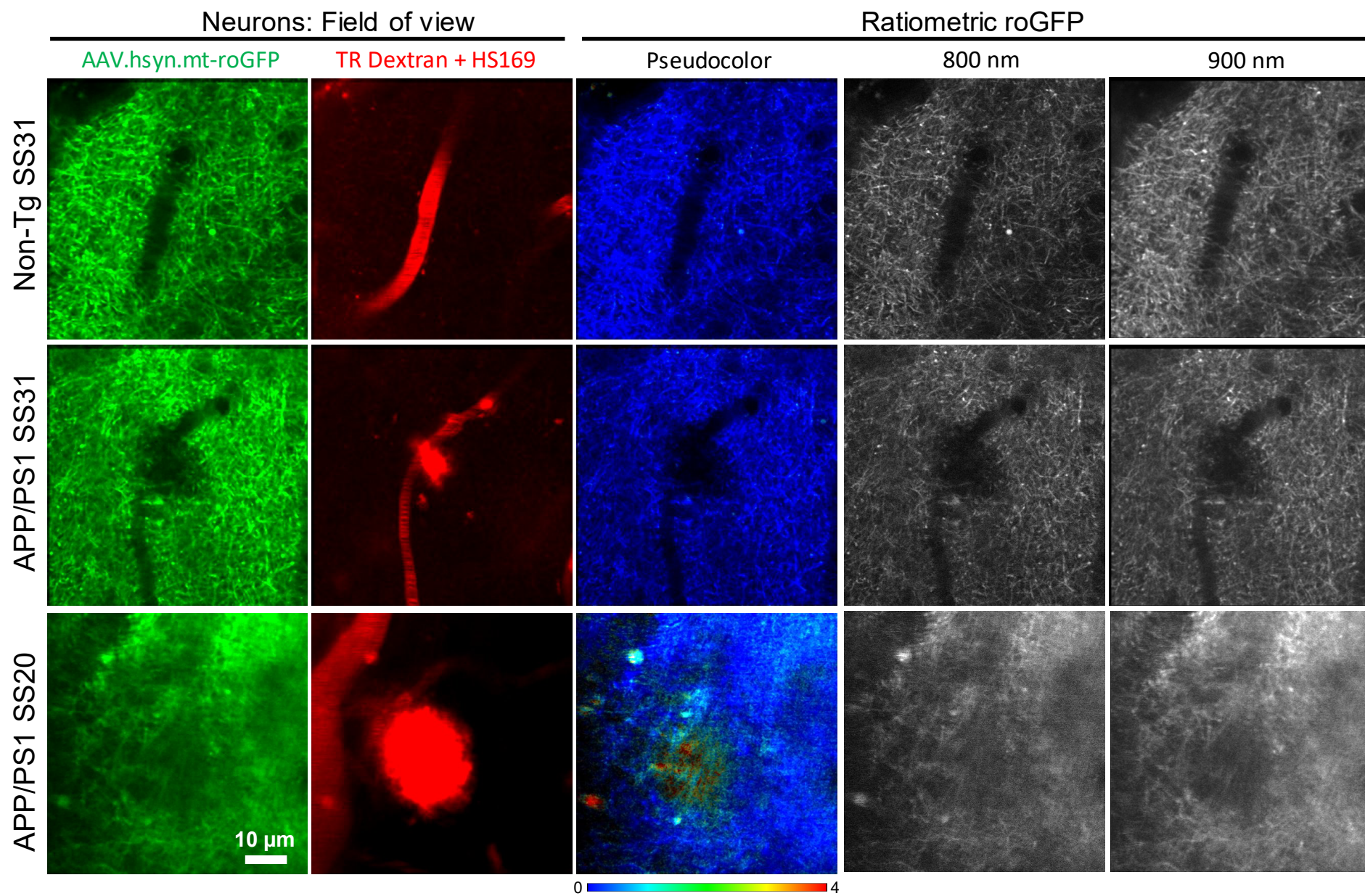

a

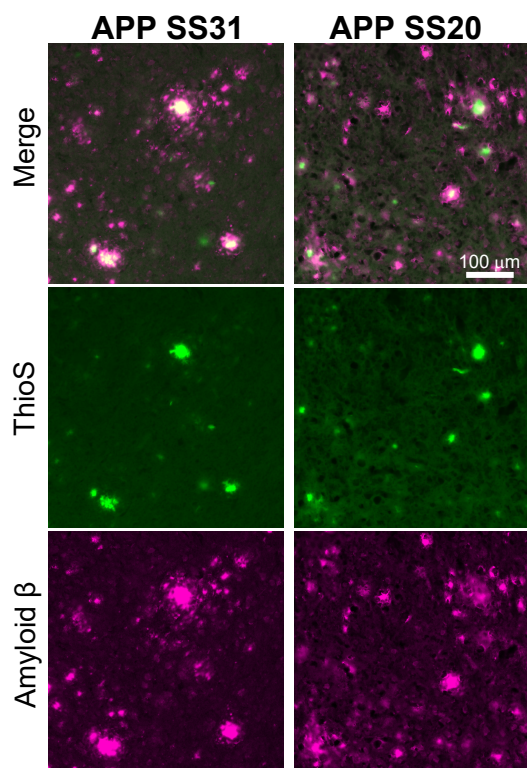

b

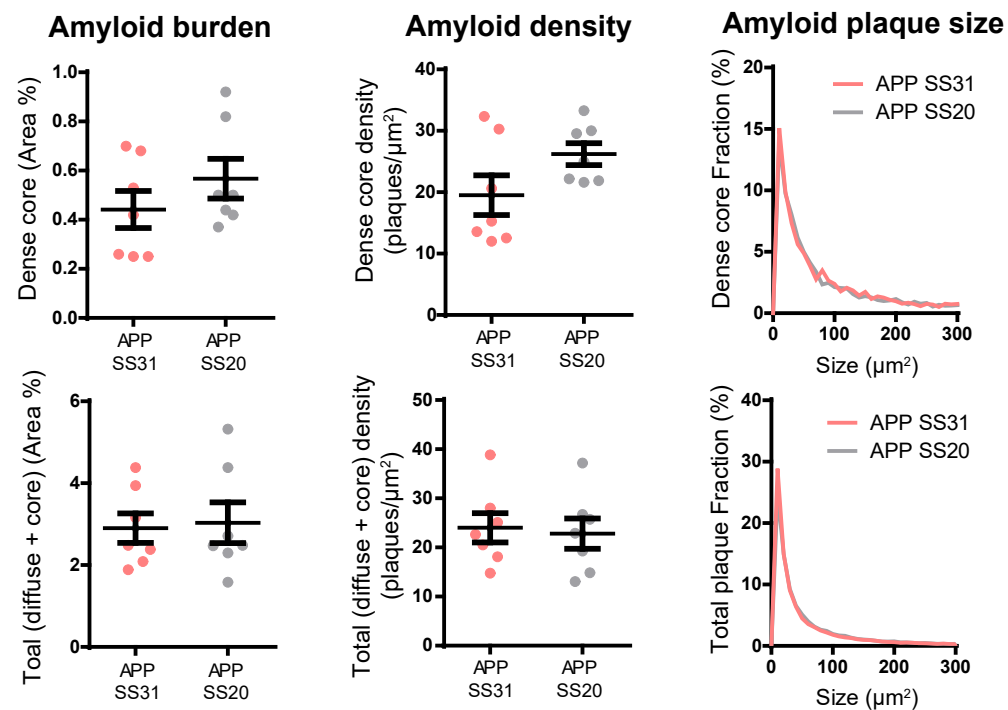

c

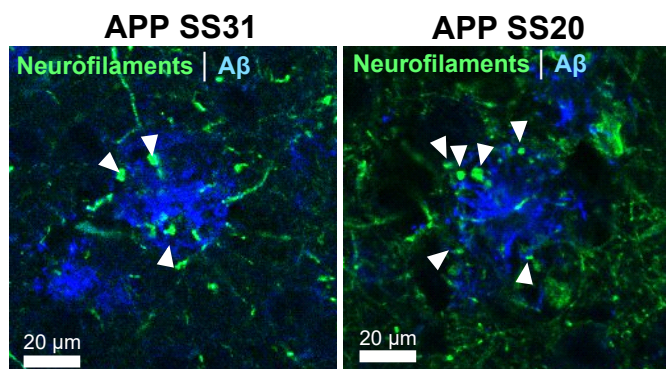

d

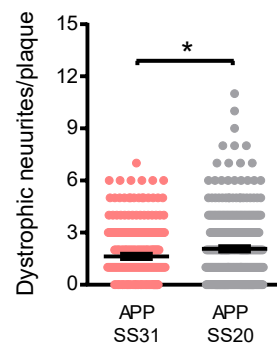

e

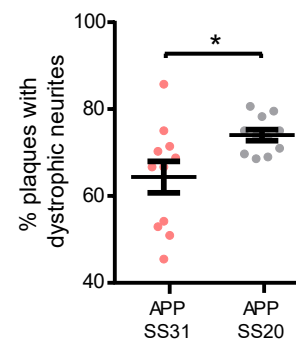

Supplement: Supplementary file 1 — Additional file 1: Fig. S1. Validation of AAV.hSyn.mt-roGFP in vitro. a. Mitochondrial co-transfection verified proper targeting of mt-roGFP to mitochondria. N2a cells (top) and primary cortical neurons (bottom) were co-transfected with mt-roGFP (green) and mRuby-Mito-7 (red) and subjected to confocal microscopy imaging. Scale bar represents 10 μm. b. Double immunolabelling of mt-roGFP (green) and mRuby-ER5 (red, targeting endoplasmic reticulum, ER) in N2a cells shows lack of colocalization and supports the mitochondrial localization of mt-roGFP. Scale bar represents 10 μm. c. In vitro imaging of cellular oxidative stress with mt-roGFP. Primary cortical neurons were exposed to either the oxidant DTDP or the reducing agent DTT. Images at 800 nm (red), 900 nm (green) and merged are shown. d. The relative changes in ratio 800/900 were represented by histograms of ratio 800/900 frequency distribution in control conditions (grey) and 20 min after exposure to DTT 1 mM (blue) and DTDP 100 μM (red) (Control, n = 143 cells; DTT 1 mM, n = 125; DTDP 100 μM, n = 109 cells). Fig. S2. Validation of pAAV.hSyn.mt-roGFP ex vivo. AAV.hSyn.mt-roGFP targets neuronal mitochondria in vivo. a. Colocalization of AAV.hSyn.mt-roGFP (green), NeuN (red) and GS (glutamine synthetase, magenta) in the mouse cortex shown by immunohistochemistry. Note that AAV.hSyn.mt-roGFP only colocalizes with the neuronal marker NeuN. Scale bar represents 10 μm. b. Colocalization of AAV.hSyn.mt-roGFP (green), HSP60 (mitochondrial marker, red) and NeuN (magenta) in the cortex shown by immunohistochemistry. Scale bar represents 10 μm. c. Inset. Colocalization of AAV.hSyn.mt-roGFP (green) and HSP60 (red) in cortex shown by immunohistochemistry (top). Scale bar 5 μm. Graph shows intensity profile of the ROI across the cell. Green line represents the fluorescence intensity of AAV.hSyn.mt-roGFP and red line represents the fluorescence intensity of HSP60. Fig. S3. Original images excited at 800nm and 900nm of Fig. 1b. [file 13024_2024_702_MOESM1_ESM.pdf]
