# Supplementary material for: Real-time imaging of mitochondrial redox reveals increased mitochondrial oxidative stress associated with amyloid β aggregates in vivo in a mouse model of Alzheimer’s disease
Source: Mol Neurodegener. 2024 Jan 18;19:6. doi: 10.1186/s13024-024-00702-2 (PMC10797952; doi:10.1186/s13024-024-00702-2)
Supplement: Supplementary file 2 — Additional file 2: Supplemental Table 1. Analysis of neuronal mitochondrial antioxidant capacity in AD vs. normal aging brain. The expression levels for genes encoding antioxidant enzymes (CAT, GLRX, GPX, GSR, GST, IDH, PRDX, ME, NNT, SOD2, TXN2, and TXNRD) were compared between control (B1, Braak NFT stages 0/I/II) and AD (B3, Braak NFT stages V/VI) individuals of a publicly available human single-nuclei RNA-seq [44]. The average expression level for each gene and group are shown, together with the log fold change and the adjusted p-value of the individual gene models, and the z-scores and the p-values of the mixed models. [file 13024_2024_702_MOESM2_ESM.docx]

| **Cell type** | **Gene** | **CTRL mean** | **AD mean** | **log FG** | **Adj. p-value** | **z-score** | **p-value** |
| --- | --- | --- | --- | --- | --- | --- | --- |
| Excitatory | IDH2 | 0.187962724 | 0.119717066 | -0.650817754 | 1.33E-133 | -10.9862994 | 4.45E-28 |
|  | PRDX5 | 0.278196733 | 0.204402998 | -0.444689124 | 1.25E-106 | -4.589778722 | 4.44E-06 |
|  | GPX1 | 0.327809697 | 0.262892894 | -0.318383387 | 1.56E-84 | -2.513403473 | 0.011957249 |
|  | ME3 | 0.434222809 | 0.400960817 | -0.114974251 | 2.54E-79 | -3.473409178 | 0.000513891 |
|  | GPX4 | 0.608532804 | 0.537161445 | -0.17997928 | 8.80E-75 | -0.77569099 | 0.437931461 |
|  | NNT | 0.468748336 | 0.433144973 | -0.113963594 | 1.92E-65 | -1.368248409 | 0.171234334 |
|  | GSTP1 | 0.160031027 | 0.12257476 | -0.384689699 | 7.86E-63 | -2.427495904 | 0.015203459 |
|  | ME2 | 0.220266036 | 0.20412805 | -0.109772612 | 2.43E-60 | -3.077360936 | 0.002088423 |
|  | IDH3A | 0.217378853 | 0.201987582 | -0.105944997 | 1.65E-56 | -2.174628513 | 0.029657966 |
|  | GSTK1 | 0.125043724 | 0.094587039 | -0.402718226 | 4.85E-56 | -3.781328855 | 0.000155993 |
|  | SOD2 | 0.495977726 | 0.463475553 | -0.09778209 | 6.64E-52 | -0.815105046 | 0.415012183 |
|  | TXN2 | 0.123506119 | 0.101184595 | -0.287592856 | 8.45E-48 | -2.877256614 | 0.004011492 |
|  | GLRX3 | 0.143234747 | 0.122711962 | -0.223105626 | 2.48E-46 | -3.07693479 | 0.00209141 |
|  | PRDX3 | 0.184419321 | 0.157731803 | -0.225516234 | 8.03E-45 | -3.182499426 | 0.001460098 |
|  | IDH3B | 0.114484131 | 0.092970305 | -0.300305742 | 2.59E-42 | -1.584722604 | 0.113029374 |
|  | GLRX2 | 0.083999028 | 0.056059075 | -0.583424683 | 4.92E-39 | -3.663770777 | 0.000248529 |
|  | ME1 | 0.401334406 | 0.421475975 | 0.070645557 | 9.49E-31 | 2.061165402 | 0.039287263 |
|  | GSTM4 | 0.058855287 | 0.047825486 | -0.299392395 | 3.67E-27 | -1.524941901 | 0.127273589 |
|  | GPX3 | 0.130465232 | 0.133474047 | 0.032893861 | 9.14E-27 | -1.358771958 | 0.174218862 |
|  | CAT | 0.049041863 | 0.041622546 | -0.236648566 | 6.34E-26 | -3.258738493 | 0.001119088 |
|  | IDH3G | 0.135656841 | 0.13651584 | 0.00910655 | 3.51E-23 | 1.229530144 | 0.218873103 |
|  | IDH1 | 0.088911333 | 0.084116426 | -0.079979772 | 1.08E-17 | 1.693496299 | 0.090361039 |
|  | TXNRD2 | 0.080438385 | 0.083008535 | 0.04537558 | 1.31E-16 | 0.358492036 | 0.719975127 |
|  | GLRX5 | 0.073134636 | 0.074988567 | 0.036115827 | 5.01E-14 | -0.039552631 | 0.968449793 |
|  | GSR | 0.115596919 | 0.13330138 | 0.205588769 | 1.16E-08 | 3.475352999 | 0.000510182 |
|  | GPX7 | 0.00795432 | 0.008010936 | 0.01023229 | 0.042385977 | 1.311758646 | 0.189601582 |
|  | GSTA1 | 0.001041579 | 0.000533536 | -0.965114434 | 0.092555287 |  |  |
|  | GSTM2 | 0.004002416 | 0.0064438 | 0.68704067 | 0.228458887 |  |  |
|  | GPX2 | 0.000687631 | 0.000505661 | -0.443463585 | 0.482749683 |  |  |
|  | GPX5 | 0.000175165 | 0.000150641 | -0.217605182 | 0.5417367 |  |  |
|  | GPX8 | 0.000583335 | 0.0014902 | 1.353107831 | 0.79809893 |  |  |
| Inhibitory | PRDX5 | 0.137349079 | 0.105359881 | -0.382521608 | 5.92E-12 | -2.804451635 | 0.005040225 |
|  | ME3 | 0.322284367 | 0.294145103 | -0.131806192 | 3.38E-10 | -0.759767805 | 0.44739339 |
|  | ME2 | 0.179491364 | 0.157975776 | -0.184211079 | 2.10E-09 | -3.530836927 | 0.000414247 |
|  | GPX4 | 0.308825688 | 0.284265835 | -0.119552044 | 3.17E-08 | 1.254054808 | 0.209822085 |
|  | GPX1 | 0.166541296 | 0.1469012 | -0.181033778 | 2.15E-07 | -0.648371591 | 0.516744641 |
|  | IDH2 | 0.084520875 | 0.067540858 | -0.323547196 | 2.44E-07 | -2.318985196 | 0.020395839 |
|  | ME1 | 0.187299032 | 0.174880737 | -0.098972053 | 1.18E-06 | -0.53022606 | 0.595955205 |
|  | IDH3A | 0.135161188 | 0.120518772 | -0.165423056 | 1.23E-06 | -1.588079196 | 0.112268432 |
|  | TXN2 | 0.070827865 | 0.054564563 | -0.376352756 | 7.98E-06 | -1.148204191 | 0.250884279 |
|  | NNT | 0.216851051 | 0.203607067 | -0.0909168 | 8.59E-06 | -1.342123145 | 0.17955607 |
|  | GSTP1 | 0.088350615 | 0.079888683 | -0.145249028 | 0.000135905 | -1.737554473 | 0.082289348 |
|  | SOD2 | 0.209429417 | 0.202938407 | -0.04542217 | 0.00039807 | -0.27648857 | 0.782172837 |
|  | GSTK1 | 0.083085775 | 0.058554529 | -0.504820723 | 0.000581944 | -1.498019964 | 0.134128064 |
|  | GLRX2 | 0.031234182 | 0.028540843 | -0.130097771 | 0.001961895 | -1.991015732 | 0.046479158 |
|  | PRDX3 | 0.090700941 | 0.079900812 | -0.182907351 | 0.005236721 | -0.001843586 | 0.998529032 |
|  | IDH3G | 0.063761515 | 0.061585306 | -0.050099719 | 0.005323398 | 0.370741751 | 0.71082989 |
|  | GLRX3 | 0.076470209 | 0.07083539 | -0.110427482 | 0.009252403 | -0.715089281 | 0.474553877 |
|  | IDH3B | 0.052541836 | 0.047424605 | -0.147830876 | 0.013509674 | 0.6399593 | 0.52219906 |
|  | GPX3 | 0.00576625 | 0.003945704 | -0.547350731 | 0.023977277 |  |  |
|  | GSR | 0.097793697 | 0.103175358 | 0.077285053 | 0.030093706 | 1.380336598 | 0.167483032 |
|  | IDH1 | 0.063948052 | 0.060452237 | -0.081104684 | 0.050863932 | 0.233630104 | 0.815272155 |
|  | CAT | 0.004618992 | 0.005930139 | 0.360488083 | 0.35199375 |  |  |
|  | GPX7 | 0.008558209 | 0.008326766 | -0.039552671 | 0.361871949 | -0.487294489 | 0.626049652 |
|  | GSTM4 | 0.029516825 | 0.028433116 | -0.053965349 | 0.463598174 | 0.61459975 | 0.538819079 |
|  | TXNRD2 | 0.040155038 | 0.050859127 | 0.3409257 | 0.469745094 | 1.756617834 | 0.078982971 |
|  | GLRX5 | 0.029054159 | 0.030839963 | 0.086056372 | 0.48121826 | 0.460349771 | 0.645265182 |
|  | GSTM2 | 0.004618745 | 0.009571672 | 1.051269942 | 0.539716633 |  |  |
| Astrocytes | IDH3A | 0.083922036 | 0.054508937 | -0.622556909 | 0.001551929 | -3.297017281 | 0.000977175 |
|  | IDH1 | 0.131711616 | 0.099576165 | -0.403510239 | 0.005924491 | -2.85945206 | 0.004243736 |
|  | CAT | 0.125268716 | 0.097248594 | -0.36527687 | 0.085835655 | -2.327388814 | 0.019944581 |
|  | IDH2 | 0.277680458 | 0.247435316 | -0.166374219 | 0.26595381 | -1.453666279 | 0.146038852 |
|  | GLRX3 | 0.060717358 | 0.047720742 | -0.347492519 | 0.338147211 | -0.66806973 | 0.504089083 |
|  | TXN2 | 0.050510664 | 0.036106945 | -0.484311653 | 0.344325586 | -0.841731762 | 0.399938115 |
|  | NNT | 0.142198687 | 0.125838081 | -0.176339564 | 0.453013958 | -1.289538402 | 0.197210975 |
|  | GLRX2 | 0.012596256 | 0.00794939 | -0.664078909 | 0.522432236 | -0.993104325 | 0.320659117 |
|  | PRDX3 | 0.028609867 | 0.029153399 | 0.027151323 | 0.522432236 | -0.769404107 | 0.441653452 |
|  | GSTM2 | 0.026151431 | 0.015720765 | -0.734218408 | 0.536901426 | -0.598809904 | 0.549299657 |
|  | IDH3B | 0.033302237 | 0.03198915 | -0.058036444 | 0.599356128 | -0.04493161 | 0.964161821 |
|  | ME1 | 0.262345239 | 0.268656146 | 0.034294232 | 0.62205411 | 1.132632199 | 0.257368741 |
|  | GPX1 | 0.032557494 | 0.027026666 | -0.268606117 | 0.63958945 | -0.459917947 | 0.645575118 |
|  | ME2 | 0.110526523 | 0.118992572 | 0.106478902 | 0.63958945 | 0.267555658 | 0.789041367 |
|  | ME3 | 0.074722806 | 0.068126841 | -0.133325329 | 0.656218031 | -0.201205762 | 0.840537686 |
|  | GSTK1 | 0.066920306 | 0.064403742 | -0.055299546 | 0.672679217 | 0.35734212 | 0.720835701 |
|  | GLRX5 | 0.010533639 | 0.01053074 | -0.000397183 | 0.800546954 | 1.147766888 | 0.25106481 |
|  | TXNRD2 | 0.034246244 | 0.036430395 | 0.089196847 | 0.810553048 | 0.989969 | 0.322189271 |
|  | GPX4 | 0.093766787 | 0.096254963 | 0.037783929 | 0.873285048 | 0.293771532 | 0.76893249 |
|  | GSR | 0.02936822 | 0.035651446 | 0.279704767 | 0.880819386 | 0.910485645 | 0.362566449 |
|  | PRDX5 | 0.071905176 | 0.066412048 | -0.114650623 | 0.907858173 | 1.287943543 | 0.197765617 |
|  | GSTP1 | 0.078824608 | 0.073320793 | -0.104423698 | 0.919884111 | 0.217805685 | 0.827580515 |
|  | GPX3 | 0.007912437 | 0.00902427 | 0.189688198 | 0.927001412 |  |  |
|  | GSTM4 | 0.034022676 | 0.037530392 | 0.141562737 | 0.929923259 | 0.958952027 | 0.337582913 |
|  | GPX7 | 0.010842843 | 0.011362537 | 0.067541958 | 0.965931931 | 0.585102953 | 0.558478493 |
|  | IDH3G | 0.031222568 | 0.027754632 | -0.169860625 | 0.970670192 | 0.382964125 | 0.701746364 |
|  | SOD2 | 0.154389485 | 0.159952644 | 0.051070336 | 0.99359659 | 1.481805644 | 0.138392017 |
| Olygod | GSTP1 | 0.078479236 | 0.09485153 | 0.273360047 | 0.000216026 | 4.726362621 | 2.29E-06 |
|  | GPX4 | 0.064471255 | 0.072938731 | 0.178029035 | 0.006160905 | 3.161555399 | 0.00156929 |
|  | IDH3G | 0.012183595 | 0.0173949 | 0.513724501 | 0.030361647 | 2.59560789 | 0.009442375 |
|  | GLRX3 | 0.029818338 | 0.033967925 | 0.187973237 | 0.1968696 | 1.836719778 | 0.066251274 |
|  | ME1 | 0.029776566 | 0.022730768 | -0.389530949 | 0.279251002 | -2.118174088 | 0.034160327 |
|  | IDH3B | 0.028213107 | 0.023240342 | -0.27973428 | 0.321138928 | -2.399811983 | 0.016403495 |
|  | SOD2 | 0.093281272 | 0.092219367 | -0.016517704 | 0.356227429 | 0.580785038 | 0.56138534 |
|  | CAT | 0.046541975 | 0.039056555 | -0.252967706 | 0.417613982 | -2.662228097 | 0.007762526 |
|  | GLRX2 | 0.009415035 | 0.011599853 | 0.301068179 | 0.436023935 | 1.098198548 | 0.2721178 |
|  | PRDX3 | 0.025037857 | 0.022722597 | -0.13998337 | 0.465892546 | 0.873361846 | 0.38246588 |
|  | IDH2 | 0.060615765 | 0.055800858 | -0.119405735 | 0.480186804 | -1.670489224 | 0.09482261 |
|  | GSTK1 | 0.058655638 | 0.052908942 | -0.148758216 | 0.51968509 | -1.787900769 | 0.073792019 |
|  | GPX7 | 0.001776977 | 0.002037291 | 0.197226564 | 0.556878645 |  |  |
|  | TXNRD2 | 0.015549666 | 0.011469205 | -0.439118241 | 0.576416453 | -1.322404107 | 0.186033622 |
|  | IDH1 | 0.026778971 | 0.029380282 | 0.13374775 | 0.640693732 | 0.220422422 | 0.825542184 |
|  | ME3 | 0.005504326 | 0.004263143 | -0.368648527 | 0.645657278 |  |  |
|  | GPX3 | 0.002859989 | 0.002074276 | -0.463401708 | 0.699894949 |  |  |
|  | GSR | 0.010850703 | 0.011877248 | 0.130412084 | 0.750870601 | 0.895107651 | 0.370729544 |
|  | IDH3A | 0.028216807 | 0.02706387 | -0.060186594 | 0.77865756 | -0.683244248 | 0.494452517 |
|  | GPX1 | 0.018134771 | 0.01610614 | -0.171147784 | 0.824495792 | -0.993521951 | 0.320455658 |
|  | GSTM4 | 0.009821928 | 0.010253107 | 0.061983008 | 0.89217432 | -0.852985501 | 0.393667345 |
|  | NNT | 0.05460491 | 0.0525784 | -0.05456038 | 0.9060949 | -0.2660739 | 0.7901823 |
|  | TXN2 | 0.02747294 | 0.02501437 | -0.135254 | 0.9158388 | -0.5944004 | 0.5522443 |
|  | ME2 | 0.0438445 | 0.04259094 | -0.04184922 | 0.9377614 | -0.8199188 | 0.4122624 |
|  | PRDX5 | 0.04387028 | 0.04113469 | -0.09288837 | 0.9674535 | 0.0611168 | 0.9512662 |
|  | GLRX5 | 0.01041129 | 0.009639698 | -0.1110884 | 0.9794574 | -0.383094 | 0.7016501 |
| Microg | GPX1 | 0.119285964 | 0.212804686 | 0.835105626 | 0.008253088 | 4.239458414 | 2.24E-05 |
|  | TXNRD2 | 0.018975673 | 0.008608761 | -1.140273503 | 0.29008051 | -1.867612013 | 0.061816171 |
|  | GPX4 | 0.03715587 | 0.062695893 | 0.754780772 | 0.389842164 | 2.056589202 | 0.039725758 |
|  | PRDX3 | 0.006642487 | 0.012881233 | 0.955475311 | 0.393893537 | 0.877840475 | 0.380030296 |
|  | NNT | 0.048206516 | 0.067733408 | 0.490639428 | 0.61568646 | 1.767495556 | 0.077145273 |
|  | IDH3A | 0.030228227 | 0.020760177 | -0.542077628 | 0.624098739 | -2.341718412 | 0.019195191 |
|  | GSTP1 | 0.026831402 | 0.038553052 | 0.522922641 | 0.630370956 | 1.910158197 | 0.056112848 |
|  | GPX7 | 0.007416482 | 0.002762821 | -1.4245927 | 0.644755628 |  |  |
|  | ME3 | 0.018861952 | 0.008392292 | -1.168342103 | 0.669456063 | -0.927976742 | 0.353419634 |
|  | CAT | 0.009362985 | 0.020590443 | 1.136934364 | 0.677525015 | 2.075174645 | 0.037970352 |
|  | PRDX5 | 0.014727712 | 0.025618629 | 0.798659979 | 0.720414928 | 1.699241153 | 0.089273755 |
|  | IDH3G | 0.011283888 | 0.015866719 | 0.491739614 | 0.743362335 | 0.555832503 | 0.578325364 |
|  | GLRX3 | 0.028557707 | 0.039276896 | 0.459800813 | 0.751613254 | 1.113826439 | 0.265353652 |
|  | ME2 | 0.10811705 | 0.120588886 | 0.157502896 | 0.775119042 | 0.426205124 | 0.669958384 |
|  | GSTK1 | 0.035875869 | 0.040066973 | 0.159399733 | 0.850169478 | 0.626598689 | 0.530922328 |
|  | TXN2 | 0.012673476 | 0.01931931 | 0.608231381 | 0.880059899 | -1.402476994 | 0.160772856 |
|  | GSR | 0.024719115 | 0.020416443 | -0.275895519 | 0.910879178 | -0.30397083 | 0.761150115 |
|  | SOD2 | 0.080023312 | 0.07219388 | -0.1485438 | 0.93720698 | -0.084689422 | 0.932508306 |
|  | ME1 | 0.014471026 | 0.019334763 | 0.418029836 | 0.949971901 | 0.227202877 | 0.820265991 |
|  | IDH1 | 0.052229913 | 0.061136869 | 0.227166372 | 0.965833717 | 0.044540398 | 0.964473651 |
|  | IDH2 | 0.064221684 | 0.057450896 | -0.160731098 | 0.965833717 | -1.102170562 | 0.270387527 |
|  | IDH3B | 0.015053242 | 0.019889641 | 0.401943012 | 0.967898967 | 0.436665204 | 0.662354168 |
|  | GSTM4 | 0.01311326 | 0.012125972 | -0.112926063 | 0.968569903 | -0.615832525 | 0.538005058 |

**Supplemental Table 1.**
